# Differential Expression of HIF1A, EPAS1, and VEGF Genes in Benign and Malignant Ovarian Neoplasia

**DOI:** 10.3390/cancers14194899

**Published:** 2022-10-07

**Authors:** Monika Englert-Golon, Małgorzata Tokłowicz, Aleksandra Żbikowska, Stefan Sajdak, Małgorzata Kotwicka, Mirosław Andrusiewicz

**Affiliations:** 1 Surgical Gynecology Clinic of The Gynecological and Obstetrics Clinical Hospital, Poznan University of Medical Sciences, Polna 33, 60-535 Poznan, Poland; 2 Department of Cell Biology, Faculty of Health Sciences, Poznan University of Medical Sciences, Rokietnicka 5D, 60-806 Poznan, Poland

**Keywords:** ovarian cancer, prognostic biomarker, hypoxia-inducible factor 1-alpha (HIF1A), endothelial PAS domain protein 1/hypoxia inducible factor 2-alpha (HIF2A/EPAS1), vascular endothelial growth factor A (VEGFA)

## Abstract

**Simple Summary:**

Ovarian cancer (OC) has the highest mortality rate of all gynecological malignancies. Moreover, at the time of the first clinical manifestation, most patients have an advanced stage of the disease. Our study examined differences in mRNA levels of hypoxia-inducible factor 1-alpha (*HIF1A*); endothelial PAS domain protein 1, also known as hypoxia-inducible factor 2-alpha (*HIF2A/EPAS1*); and vascular endothelial growth factor A (*VEGFA*) between cancerous tissue, benign hyperplastic changes in the ovary, and normal tissue. We found that gene expression changes were visible not only in the case-control study, but also along with changes in severity. We observed disturbances in the expression levels of interdependent genes. Our findings suggest that mutual association in the expression of both *HIF1A* and *HIF2A/EPAS1* with *VEGFA* has prognostic importance for patients with OC. Our observations may help identify patients for clinical trials aimed at inhibiting the hypoxia-induced neovascularization-dependent pathways.

**Abstract:**

Ovarian cancer (OC) has the highest mortality rate of all gynecological malignancies. Moreover, at the time of the first clinical manifestation, most patients have an advanced stage of the disease. Our study examined differences in mRNA levels of hypoxia-inducible factor 1-alpha (*HIF1A*); endothelial PAS domain protein 1, also known as hypoxia-inducible factor 2-alpha (*HIF2A/EPAS1*); and vascular endothelial growth factor A (*VEGFA*) between cancerous tissue, benign hyperplastic changes in the ovary, and normal tissue. Our cohorts consisted of 52 patients diagnosed with OC (*n* = 55), benign non-cancerous changes (*n* = 21), and normal tissue samples (*n* = 38). The mRNA expression level was evaluated using RT-qPCR. We found that gene expression changes were visible not only in the case-control study, but also along with changes in severity. Additionally, the gene expression was differentiated in age, BMI, menopausal status, and the number of comorbidy-related groups. Furthermore, our findings demonstrate that analyzing the correlation between genes is essential. In a case-to-case and case-to-control study, we observed disturbances in the expression levels of interdependent genes. Our findings suggest that mutual association in the expression of both *HIF1A* and *HIF2A/EPAS1* with *VEGFA* has prognostic importance for patients with OC. Our observations may help identify patients for clinical trials aimed at inhibiting the hypoxia-induced neovascularization-dependent pathways.

## 1. Introduction

Epithelial ovarian cancer (OC) has the highest mortality rate of all gynecological malignancies [1], with most patients presenting with advanced disease. Furthermore, it is common to find metastatic foci outside the pelvis to the peritoneum, reflecting the aggressive nature of the malignancy [2,3]. It brings added complications of malignant ascites and impaired bowel movements, severely impacting quality of life and survival [4,5]. This peritoneal invasion is characterized by cancer cells detaching from the primary tumor and colonizing and expanding within the peritoneum. At this point, cancer cells may be susceptible to hypoxia [6,7,8]. The ability of cancer cells to adapt to lower oxygen saturation is critical to their survival in both the original foci and new cavities. The occurrence of hypoxia is a well-known phenomenon that correlates in many tumors with poor prognosis [9,10,11,12]. These tumor cells that survive in adverse environmental conditions show gene expression and structural changes.

Interestingly, hypoxia may be a cause or effect of differential gene expression in pathologically altered tissue. Thus, elevated or depressed levels of glucose transporters, glycolic pathway enzymes, oxygenases, and other genes have been observed [9,13]. On the other hand, neovascularization and angiogenesis are known attributes of cancer, including ovarian malignancies [14]. Angiogenesis is known as the formation of new blood vessels from the existing vasculature. It promotes cancer progression and has been demonstrated to strongly correlate with the risk of invasion and metastasis [15,16]. Vascular endothelial growth factor (VEGF) is a known pro-angiogenic factor that stimulates angiogenesis [15]. An elevated VEGF level in serum and tissue has been reported in OC patients and was associated with a poor prognosis [17,18]. However, recent advances have been made in ovarian cancer therapies targeting anti-angiogenic genes and proteins in the cancer environment [19]. Additionally, the VEGFA presence was reported in human breast cancer specimens [20]. Moreover, its elevated level was also related to more aggressive mammary carcinoma subtypes in animal models. VEGFA and its receptors’ signaling pathways could be an alternative to the hypoxia mechanism leading to cancer progression. As a result, it suggests that VEGFA could be a suitable diagnostic biomarker for, e.g., mammary carcinoma [21].

The balance between the stimulating and inhibitory effects of angiogenic factors modulates angiogenesis. An imbalance favoring angiogenesis occurs during the initial formation of a tumor mass and accompanies the metastatic progression and spread of cancer cells [22]. Hypoxia is believed to be a key factor resulting in the increase of VEGF expression [23,24]. Numerous mechanisms regulate hypoxia-induced VEGF expression, one of which is hypoxia-inducible factor 1 (HIF1)-dependent transcriptional activation. HIF1 is a heterodimeric transcription factor composed of two subunits, namely the alpha and beta chains (HIF1A and HIF1B, respectively) [25,26]. The heterodimeric (alpha/beta) HIF1A complex binds DNA and induces genes relevant to tumor progression, e.g., those responsible for angiogenesis or metastasis control [6,27,28]. Three isoforms of HIF1 alpha have been characterized, and the best described are HIF1A and HIF2A (called endothelial PAS domain protein 1 (EPAS1)). It has been reported that both isoforms show different biological activity during embryogenesis [19,26], and a diverse hypoxia response depends on the expression of both isoforms in various tumor types [6,29,30,31]. Although sequence similarity of HIF1A and EPAS1 reaches approximately 50%, they regulate diverse targets due to their different transactivation domains [32,33,34]. In addition, HIF1A is widely expressed, while EPAS1 is only expressed in certain cell types [34,35,36]. In vitro findings suggest that nuclear HIF1A has prognostic importance in ovarian cancer, and EPAS1 may play a crucial role in the carcinogenesis and progression of ovarian malignancies [37].

Our study examined any potential differences in mRNA levels of HIF1A, HIF2A/EPAS1, and VEGFA mRNA within cancerous tissue, benign ovarian changes, and normal tissue. Furthermore, we analyzed relevant correlations between expression levels and pathological grade. We examined whether other factors, and not only ovarian tissue related factors (such as menopausal status, comorbidities presence, age, and BMI), influence changes in gene expression. Previous studies have demonstrated that HIF1A is a potential target for cancer therapies [27,38]. Our study’s results may help identify ovarian carcinoma patient subgroups and whether they could be potential candidates for clinical trials examining HIF1 pathway-targeted therapies.

## 2. Materials and Methods

### 2.1. Ethics

The study was conducted according to the guidelines of the Declaration of Helsinki and approved by the IRB of Poznan University of Medical Sciences (PUMS protocol code Nos. 46/12; date of approval 1/5/2012, and 593/19, and 594/19; date of approval 6/19/2019). Written informed consent was obtained from all study participants.

### 2.2. Patient Demographic Data

Between January 2017 and December 2020, 114 women underwent surgery at the Surgical Gynecology Clinic of the Gynecological and Obstetrics Clinical Hospital, Poznan University of Medical Sciences. The study cohort consists of patients with diagnosed ovarian carcinoma (*n* = 55). Normal tissue samples that lack cancerous changes (examined as described before [39]) were obtained from patients who underwent a total hysterectomy (*n* = 59). The absence or presence of cancerous changes was confirmed by anatomicopathologic macroscopic and intraoperative microscopic examinations. No patients received chemotherapy or radiotherapy prior the surgery. The CA125 and HE4 markers’ serum level was determined in all patients. Operative findings determine the precise histologic diagnosis and stage [39].

All women were of Caucasian descent, and patient characteristic is shown in Table 1. Tissue samples were immersed in an RNA-protective medium [40] and processed at the Chair and Department of Cell Biology, PUMS, or stored at −80 °C until RNA isolation could be performed.

**Figure 1:**
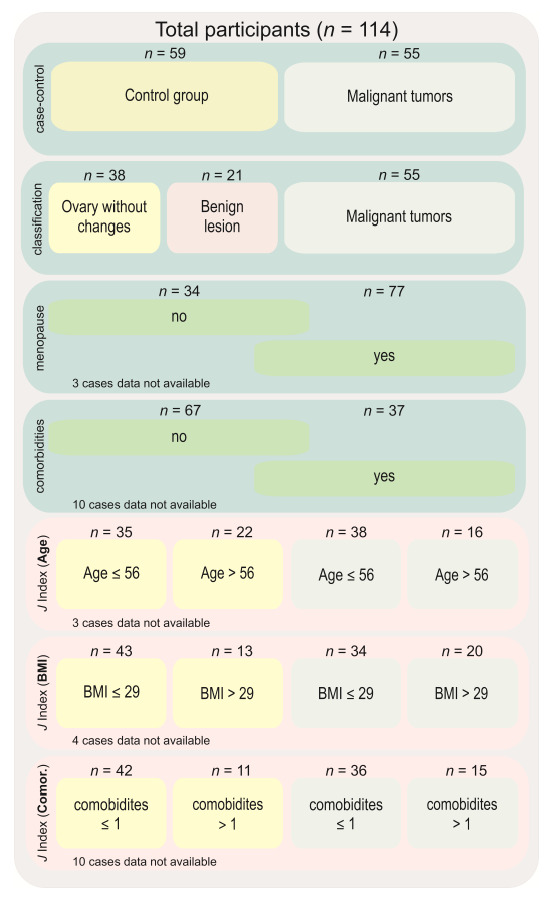
Diagram showing groups of comparisons carried out in this study. Abbreviations: *n*—number of cases, *J* index—Youden’s *J* statistic, BMI—body mass index.

Expression levels of *HIF1A*, *HIF2A*/*EPAS1*, and *VEGFA* were analyzed in different groups. First, we compared controls to OC cases. Subsequently, the control group was divided into ovary samples without any pathological changes and those with a benign, non-cancerous lesions. We then compared those two subgroups with the results from the malignant tissue. Gene expression was also analyzed in subgroups related to menopausal status and the presence of comorbidities (regardless of the cancer manifestation). The last analysis covered the cut-off points based on receiver operating characteristics (ROC) and Youden’s *J* index for age, body mass index, and comorbidities (Figure 1).

### 2.3. Methods

#### 2.3.1. Nucleic Acid Extraction and Validation

High molecular weight RNA, microRNA fraction-free, was extracted from tissue specimens using the microRNA and RNA isolation kit according to the manufacturer’s protocol (A&A Biotechnology, Gdynia, Poland) as described previously [39]. The quality, quantity, and purity of obtained RNA were analyzed spectrophotometrically as described previously [41], and RNA integrity was evaluated by agarose gel electrophoresis in denaturing conditions [41].

#### 2.3.2. Reverse Transcription and Quantitative PCR

**Figure 2:**
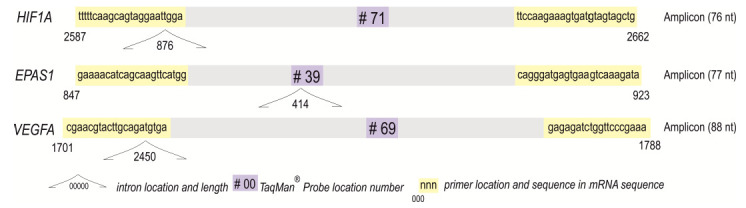
Amplicons of analyzed self-designed assays for *HIF1A*, *EPAS1/HIF2A*, and *VEGFA* with the primers, TaqMan^®^ Probes, and introns positions.

Complementary DNA was synthesized following the Transcriptor Reverse Transcriptase manufacturer’s protocol (Roche, Basel, Switzerland) in a total volume of 20 μL [39,41]. Using the LightCycler 2.0 carousel glass capillary-based system (Roche, Manheim, Germany), the relative mRNA levels of *HIF1A* (NCBI GenBank Reference Sequence: NM_001530.4 and NM_001243084.2, transcript variants 1 and 3, respectively), *EPAS1*/*HIF2A* (NM_001430.5), and *VEGFA* (NM_001025366.3, NM_003376.6, NM_001025367.3, NM_001025368.3, NM_001287044.2, transcript variants 1–4 and 10, respectively) were established. Primer sequences and TaqMan hydrolysis probe positions for the gene of interest (GOI) were determined using Universal ProbeLibrary (UPL) Assay Design Center algorithm (http://qpcr.probefinder.com, accessed on 28 September 2017). The TaqMan locked nucleic acid probes #71 (cat. no. 04688945001), #39 (cat. no. 04687973001), and #69 (cat. no. 04688686001) are commercially available (Roche, Basel, Switzerland). The description and location of probes and primers (with sequences) for the self-designed assays are shown in Figure 2. The hypoxanthine-guanine phosphoribosyltransferase (*HPRT*) gene assay (cat. no. 05532957001; Roche, Basel, Switzerland) served as an internal control [39,41].

Quantitative polymerase chain reactions’ cycling and acquisition steps, standardized for Roche UPL hydrolyzing probes, were conducted as described before in a total volume of 20 μL [39,41,42,43]. Each reaction was performed in duplicate on independently synthesized complementary DNA, and the mean values were used for statistical analyses. Reaction efficiencies were obtained from standard curves [41]. Threshold values raw data were analyzed by comparing them to appropriately selected standard curves and reference gene assay using LC 5.0.0.38 software and presented as concentration ratio (Cr).

#### 2.3.3. Statistical Analyses

Statistical analyses were performed using Statistica^®^ Version 13.5.0 software for Windows (TIBCO Software Inc., Palo Alto, CA, USA) and PQStat 1.8.0.414 software (PQStat software; Poznan, Poland). The ROC curve and Youden’s *J* statistic (Youden’s index) were used to analyze the discriminatory ability to distinguish between populations. ROC curve enabled selecting an optimal threshold value (cut-off point) for age, BMI, and the number of comorbidities for subgroup selection. Patients’ results were compared in groups. The GOIs concentration ratio values were rescaled for each gene separately, using the min-max formula.

$${\mathrm{Cr}}^{\mathrm{norm}}=\frac{\mathrm{Cr}-\min\left(\mathrm{Cr}\right)}{\max\left(\mathrm{Cr}\right)-\min\left(\mathrm{Cr}\right)}.$$

where Cr is the concentration ratio, Cr^norm^ is the normalized Cr value, and max(Cr) is the minimal and maximal Cr value.

The Shapiro–Wilks test was used to assess data normality. A two-sided Mann–Whitney U test and a Kruskal–Wallis test were used with Dunn’s post hoc test for normal data. A Bonferroni–Hochberg correction was used to test multiple comparisons. Spearman’s rank correlation tests determined the correlation coefficient (R) between parameters. Data were considered statistically significant at *p* < 0.05.

## 3. Results

### 3.1. Case-Control Study

In control and malignant carcinoma patients, a significant age difference was observed, with patients presenting cancer being significantly older (*p* = 0.0072; means ± standard deviations: 54 ± 12.4 yrs vs. 59 ± 10.4 yrs). However, both groups did not differ in BMI or the number of comorbidities present (*p* > 0.05).

**Figure 3:**
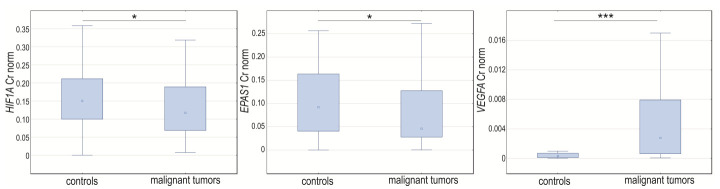
Box-whiskers plot of *HIF1A*, *EPAS1*, and *VEGFA* normalized expression level in controls and ovarian cancer patients. Cr norm—normalized concentration ratio. * *p* < 0.05; *** *p* < 0.001.

In the case-control study, we noticed changes in the normalized expression of all analyzed genes. The normalized expression levels of *HIF1A* and *EPAS1* were significantly higher in control samples (*p* = 0.04204 and *p* = 0.0118, respectively) and *VEGFA* was lower (*p* < 0.0001) compared to cancerous changed tissue (Figure 3).

Subsequently, after examining differences in the expression level of genes of interest (GOI) between both analyzed groups, the Spearman rank correlation coefficient for the normalized concentration ratio was established. First, we analyzed whether the expression of the targeted genes was correlated within the whole group, irrelevant to the case-control study. We found that *HIF1A* and *EPAS1* were positive and moderately correlated (R = 0.47, *p* < 0.0001). Expression of *HIF1A* was also significantly and positively but weakly correlated with *VEGFA* (R = 0.25, *p* = 0.0077). Subsequently, we analyzed the correlation in the case-control groups. In controls, all analyzed GOIs expression levels were significantly and positively correlated. *HIF1A* with *VEGFA* correlated moderately (R = 0.53, *p* < 0.0001) and *EPAS1* with both *HIF1A* and *VEGFA* correlated weakly (R = 0.26, *p* = 0.437 and R = 0.36, *p* = 0.0056, respectively). On the other hand, in ovarian cancer tissue samples, *VEGFA* was correlated neither with *HIF1A* nor *EPAS1*, but *HIF1A* was positively and moderately correlated with *EPAS1* (R = 0.57, *p* < 0.0001) (Supplementary Materials, Table S1).

### 3.2. Classification Due to Intensification of Pathological Changes

We assigned controls into two subgroups (cases without changes and benign non-cancerous lesions) and compared them to women with cancer. We observed age differences between the groups (*p* = 0.0173). Although there were no significant differences in age between controls without any changes and the other groups (*p* > 0.05), the cancerous group was significantly older than those with benign changes (*p* = 0.0269).

**Figure 4:**
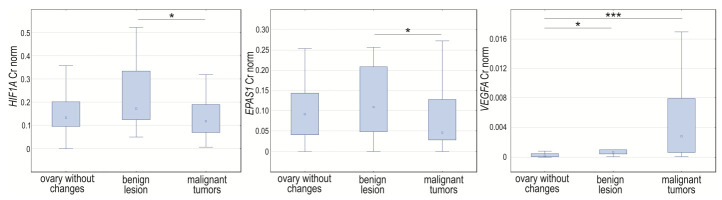
Box-whiskers plot of *HIF1A*, *EPAS1*, and *VEGFA* normalized expression level in controls, women with benign non-cancerous changes, and ovarian cancer patients. Cr norm—normalized concentration ratio; * *p* < 0.05; *** *p* < 0.001.

Analyzing the *HIF1A*, *EPAS1*, and *VEGFA* expression, we noticed changes in normalized expression in tissue samples assigned due to intensification of pathological changes into three groups (*p* = 0.0448, *p* = 0.0341, and *p* < 0.0001, respectively). The *HIF1A* and *EPAS1* expression levels were higher in benign lesions compared to malignant tumors (*p* = 0.04067 and *p* = 0.0228, respectively). The normalized expression level of *VEGFA* was significantly lower in unchanged tissues when compared to cancerous tissue (*p* < 0.0001) and samples with milder changes (*p* = 0.0138) (Figure 4).

The significant Spearman rank correlation coefficients for the normalized concentration ratios of GOIs demonstrated a positive but weak correlation between *HIF1A* and *EPAS1* (R = 0.32, *p* = 0.0474), a strong correlation in the case of *HIF1A* and *VEGFA* (R = 0.71, *p* < 0.0001), and a moderate correlation for *EPAS1* and *VEGFA* (R = 0.45, *p* = 0.0045) in non-changed tissue samples. In tissue classified as benign ovarian changes, the GOIs were not significantly correlated (*p* > 0.05) (Supplementary Materials, Table S2). The cancer tissue correlations were described in the case-control study (Supplementary Materials, Table S1).

### 3.3. Menopausal Status

As expected, when considering comorbidities, the women suffered more diseases post-menopause (*p* = 0.0003), and the occurrence of comorbidities was higher (*p* = 0.0071; risk ratio = 3.32, 95% confidence interval [1.36–8.12]). There was no difference between the groups in BMI (*p* > 0.05).

**Figure 5:**
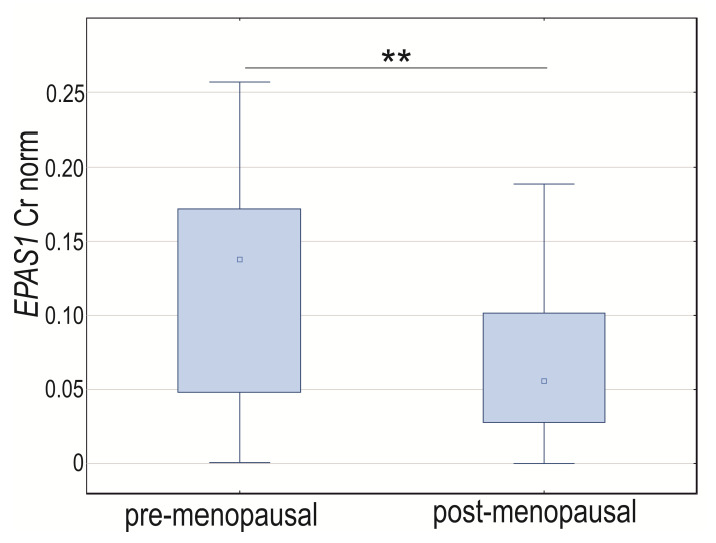
Box-whiskers plot of *EPAS1* normalized expression level in women before and after menopause. Cr norm—normalized concentration ratio; ** *p* < 0.01.

Analyzing the expression of GOI based on menopausal status, we did not notice changes in *HIF1A* and *VEGFA* normalized expression between groups (*p* > 0.05). The *EPAS1* mRNA level was elevated in tissues obtained from women before menopause (*p* = 0.0022; Figure 5). The Spearman rank correlation coefficient for normalized concentration ratio showed a positive and moderate correlation of *HIF1A* and *EPAS1* (R = 0.50, *p* < 0.0001) in the post-menopausal group and *HIF1A* and *VEGFA* in the women before menopause (R = 0.47, *p* = 0.0053). Neither *HIF1A* nor *EPAS1* was significantly correlated with *VEGFA* in menopausal women nor *EPAS1* with *HIF1A* and *VEGFA* in pre-menopausal women (*p* > 0.05) (Supplementary Materials, Table S3).

### 3.4. Comorbidities Presence

Comparing women with the presence or absence of comorbidities (hypertension, diabetes, thyroid disease, varicose veins, stroke, heart disease, varicose veins, COPD, psoriasis, myasthenia gravis, ulcerative colitis, and asthma), there were significant age differences and BMI observed (*p* < 0.0001). The cases with comorbidities were older and had a higher body mass index.

**Figure 6:**
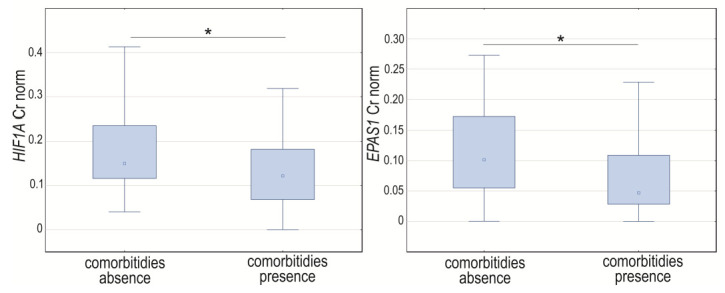
Box-whiskers plot of *HIF1A* and *EPAS1* normalized expression level in cases with comorbidities presence or absence. Cr norm—normalized concentration ratio; * *p* < 0.05.

Analyzing GOIs’ expression, we noticed changes in the normalized expression between groups with either the presence or absence of comorbidities for *HIF1A* (*p* = 0.0255) and *EPAS1* (*p* = 0.0423). The expression level of these genes was significantly higher in the group without comorbidities (Figure 6). The Spearman rank correlation coefficient for normalized concentration ratio showed a positive and moderate or weak correlation between *HIF1A* and *EPAS1* in cases with and without comorbidities (R = 0.40, *p* = 0.0007, and R = 0.34, *p* = 0.0404, respectively). Neither *HIF1A* nor *EPAS1* significantly correlated with *VEGFA* (*p* > 0.05), independent of the presence of comorbidities (*p* < 0.05) (Supplementary Materials, Table S4). Comorbidities number was not significantly correlated with analyzed GOIs expression level (*p* > 0.05).

### 3.5. Cut-Off Points of Age, BMI, and Comorbidities Number

**Figure 7:**
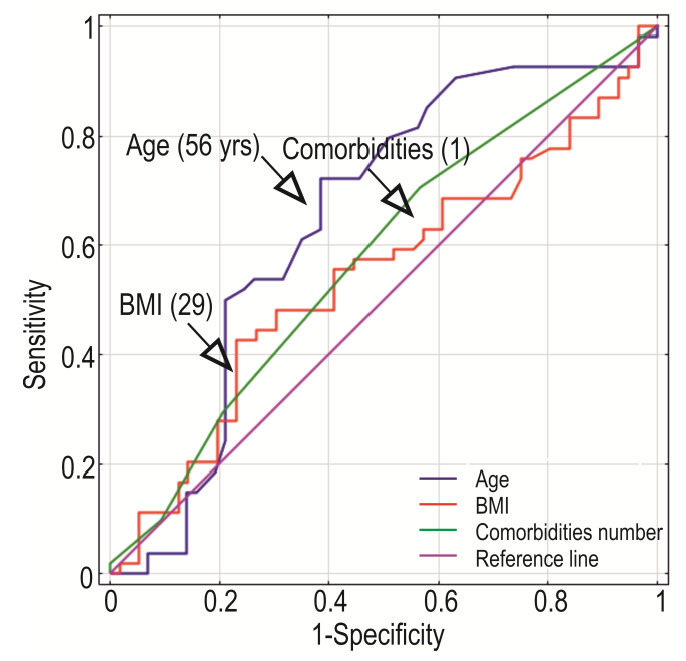
Receiver operating characteristics for age, BMI, and comorbidities in a case-control study. Based on Youden’s *J* Index, the cut-off points were estimated and showed as values in the brackets.

We used Youden’s *J* statistic to determine the division of the studied controls and cases using cut-off points for age, BMI, and the presence of comorbidities. The cut-off values were as follows: for patients age = 56 years, body mass index BMI = 29, and comorbidities = 1 (Figure 7).

Analyzing the GOI expression when subgroups were based on the Youden’s *J* statistic for age, we observed significantly elevated levels of both *HIF1A* (*p* = 0.0023) and *EPAS1* (*p* = 0.0246) between controls and cancer patients in the group > 56 yrs. The *VEGFA* level was significantly reduced in both age groups, >56 yrs (*p* = 0.0299) and ≤56 yrs (*p* < 0.0001). Additionally, significantly higher expression was observed between groups distinguished by Youden’s *J* cut-off points for age, in the cancer group, for *HIF1A* (*p* = 0.0398) and *EPAS1* (*p* = 0.0022) (Supplementary Materials, Figure S1).

When analyzing GOIs’ expression between controls and cancer patients in the division based on the Youden’s index regarding BMI, the expression of *VEGFA* was significantly higher in malignant lesion samples in the group with a BMI ≤ 29 (*p* < 0.0001) and *EPAS1* in controls in the group BMI > 29 (*p* = 0.0084). Additionally, *EPAS1* expression was elevated in cancer patients with ≤29 BMI compared to BMI > 29 (*p* = 0.0118) (Supplementary Materials, Figure S1).

Analyzing GOI expression in divisions distinguished based on the Youden’s index regarding comorbidities number, the normalized expression level of *EPAS1* and *VEGFA* differed significantly (*p* = 0.0133 and *p* < 0.0001, respectively) between controls and cancer patients, but only in group ≤ 1 was there comorbid disease presence, and in cancerous tissue they were lower for *EPAS1* and higher for *VEGFA* (Supplementary Materials, Figure S1).

## 4. Discussion

Peritoneal invasion of ovarian cancer is characterized by cancer cells detaching from the primary tumor foci, colonizing new habitats, and expanding in the peritoneum. Thus, while cancer cells may be susceptible to hypoxia [6,7], new blood vessels are formed from the existing vasculature to avoid oxygen deficiency [14]. It promotes cancer progression and strongly correlates with the risk of invasion and metastasis [15,16]. Changes in the expression level of different genes and their mutual control and linkage could help cancer cells survive in unfavorable environmental conditions [44].

Our investigations examined changes in expression levels of the main signaling pathway’s core components that enable cancer cells to adapt and survive in the poorly oxygenated microenvironment of solid tumors. The *HIF1A* and *EPAS1* serve as an angiogenic master switch for *VEGFA* gene regulation, which is essential for physiological and pathological angiogenesis [45,46,47]. We found that expression of *HIF1A* and *EPAS1* was higher in controls and *VEGFA* higher in patients with malignant tumors. Additionally, *HIF1A* and *EPAS1* mRNA levels were significantly higher in benign non-cancerous lesions than in malignant lesions, but not in control samples. In turn, *VEGFA* expression was significantly higher in malignant tumors compared to control tissues, and there was a difference in *VEGFA* expression between benign lesions and normal ovarian tissue. The mRNA level was the lowest in the ovary without changes. Other authors described *HIF1A* elevated expression in stage III and IV ovarian cancer compared to controls. It was shown that stage III was characterized by the highest expression [48]. Other authors, in turn, have not observed differences in *HIF1A* expression and demonstrated similar cytoplasmic protein content in early and advanced tumor stages.

Conversely, the same team showed that nuclear expression of *HIF1A* and higher cytoplasmic levels of *EPAS1* might be associated with OC progression [6]. In ovarian carcinoma, elevated expression of *HIF1A* alone is not considered a prognostic marker [49]. However, it has been reported that the level and cellular localization combined, which could be hypoxia dependent, may have prognostic and diagnostic potential in ovarian cancer [6]. Additionally, the activation of *HIF1A* and *EPAS1* in cancer is connected with the vascularization processes, and ovarian carcinoma cells have been shown to express elevated *VEGFA* levels [50]. Moreover, an elevated level of *VEGFA* was found to be a key molecule in ovarian cancer [17,51,52]. Still, other hypoxia and angiogenic factors are needed to be found to clarify the mechanism [6]. Our results regarding increased *VEGFA* expression level in malignant tumors compared to controls and benign lesions are supported by other authors. They showed an increased level of the VEGFA protein associated with the ovarian tumor stage [48]. Finally, *VEGFA* expression in OC cells has been considered a poor prognostic factor as it exerts an influence on tumor immune evasion via the recruitment and activation of myeloid-derived suppressor cells [53,54]. We established that the expression level of all GOIs examined herein demonstrated mutual positive correlations in normal tissue.

Contrary to this, in cancerous tissue, only *HIF1A* level moderately correlated with *EPAS1*. The correlations were similar to the whole control group in tissue samples lacking any pathological changes. On the other hand, we did not observe any corresponding correlations in the benign lesion. Thus, it seems that along with the increasing level of tissue pathology, there is a greater disturbance in these genes’ control and mutual relations. Disturbed gene expression correlations could be explained by mechanisms other than hypoxia that influence the *VEGFA* expression, such as cytokines or growth factors [48]. As we also established, age, BMI and menopausal status, or comorbidities may influence the analyzed gene expression pattern.

In our research, menopausal status and the comorbidities’ presence affect the expression level of analyzed genes. The *EPAS1* mRNA level was elevated in tissues obtained from women before menopause. Positive and moderate *HIF1A* with *EPAS1* in the post-menopausal group and *HIF1A* with *VEGFA* correlation in the women before menopause were shown. Additionally, we noticed higher *HIF1A* and *EPAS1* levels in the cases without comorbidities. We also observed a moderate *HIF1A* and *EPAS1* correlation in women after menopause and in both control groups with and without comorbidities. Thus, menopausal status could influence the genes examined herein, and other diseases affect the expression.

Nevertheless, data regarding HIF1A, EPAS1, and VEGFA expression in ovarian cancer are scant. A little more is known about their expression in other cancers. However, while hypoxia is generally associated with poor prognosis, the prognostic role of HIF1A and EPAS1 differs between tumor types. In some cancers, e.g., head and neck, lung, and neuroblastoma, a higher EPAS1 level was associated with a better prognosis [55,56,57]. It was also shown that the HIF1A and EPAS1 proteins’ cellular localization could be related to the further prognosis of the patients [46]. The VEGFA presence was reported in non-neoplastic breast specimens. The expression of different *VEGFA* isoforms was significantly higher in pre-menopausal than post-menopausal women and was negatively correlated with age.

On the contrary, it was shown that *VEGFA* mRNA expression levels decrease after menopause in normal breast tissue but not in breast cancer lesions [58]. Moreover, it has been described that *VEGFA* expression was not only affected by menopausal status and BMI, but among perimenopausal women with cardiometabolic diseases, combined with obesity, the polymorphic changes in the *VEGFA* influence its expression [59]. The differential gene associations could partly explain the varying expression level. Our results showed that in controls, expression levels of all GOIs correlated, but not in benign lesions.

This observation could be supported by further analyses performed in groups segregated by Youden’s index. The GOIs expression pattern differed in the case-control study. The *HIF1A* and *EPAS1* mRNA levels were higher in controls aged >56 years, while *VEGFA* was higher in patients with malignant changes in both age groups. Moreover, between distinguished age groups, cancer cases differed in EPAS1 and HIF1A. The mRNA level of both genes was higher in younger cancer patients. Regarding BMI groups segregated by Youden’s index, there was no difference between the distinguished groups in *HIF1A* expression level. Considering malignant changes, we established lower *EPAS1* mRNA levels compared to controls in the group with higher BMI values. Additionally, in BMI ≤ 29 group, the *VEGFA* mRNA level was higher in tumor samples. It was shown that inflammatory mediator levels increase in obesity and have been shown to regulate *HIF1A* levels in adipose tissue. This obese tissue is recognized as more hypoxic than healthy adipose tissue, a major driver of HIF1A stabilization. It is likely that obesity-associated factors, including leptin, converge to regulate metabolic pathways leading to a hormonal milieu conducive to tumor growth [60].

Expression differences were observed in the case of *EPAS1* and *VEGFA* between groups distinguished based on comorbidities manifestation. We showed *EPAS1* elevated level in controls and *VEGFA* in malignant tumors, but only in the group with a lower number of comorbidities. Our observations are consistent with other authors. Although other authors have analyzed the correlation of protein expression patterns, all of the observed correlations were positive, as in our case [6]. It has been shown that ovarian carcinoma cells presented higher VEGFA protein content, and HIF1A level was significantly correlated with VEGFA [50]. The age-related altered expression of *VEGFA* remains in line with observations in other malignancies and could also be associated with microRNA targeting of its transcripts [61,62].

Additionally, VEGFA upregulated by HIF1A and/or EPAS1 genes might be involved in some OC patients’ angiogenesis [63]. It was shown that, compared with benign ovarian tumor tissue, malignant cancer tissues demonstrated a significantly higher expression of VEGFA, which is considered an unfavorable prognostic factor [53,64]. It has been demonstrated that *VEGFA* isoforms positively correlated with tumor biology and were co-expressed in ovarian cancer as indicators of tumor activity [65]. The expression of analyzed genes could be related to many factors, such as age, obesity, or comorbidities, or be independent prognostic factors and therapeutic targets.

## 5. Conclusions

Our findings strongly suggest that mutual relationships exist in the expression of both HIF1A and EPAS1 with VEGFA, which has prognostic importance for malignant ovarian carcinoma. Upregulation of VEGFA plays an important role in the oncogenesis and progression of ovarian carcinoma. Over the past years, HIF1A [26,38], EPAS1 [37,66,67], and VEGFA [68,69,70] have been highlighted to be attractive potential targets for anti-cancer therapies. Anti-VEGFA therapy in ovarian cancer has already been confirmed to improve the effectiveness of standard treatment [71]. Our findings allow us to identify patients as potential candidates for clinical trials aimed at inhibiting the hypoxia-induced neovascularization-dependent pathways.

## Figures and Tables

**Table 1:** Table showing patient characteristics.

Characteristics	*N* (%)
Total	114
Age (in years)	
Mean	57
Median	58
Range	25-81
BMI	Controls/cases
Underweight	2/2
Normal	27/21
Overweight	16/17
Obese (classes I, II, and III)	11/14
Case-control	55/59
Control ovaries	59 (52)
Malignant tumors	55 (48)
Classification	
Ovary without changes	38 (33)
Benign ovarian lesion	21 (19)
Malignant ovarian tumors	55 (48)
Histopathological grades of ovarian tumors	Available in 45/55 patients
G1	5 (9)
G2	2 (4)
G3	38 (69)
N/A	10 (18)
FIGO	Available in 48/55 patients
IA	2 (4)
IB	2 (4)
IC	9 (16)
II	1 (2)
IIIA	5 (9)
IIIB	7 (13)
IIIC	17 (31)
IV	5 (9)
N/A	7 (13)
Histology	Available in 55/55 patients
Adenocarcinoma serosum	38 (69)
Adenocarcinoma mucinosum	5 (11)
Adenocarcinoma clarocellularae	4 (7)
Adenocarcinoma endometrioides	3 (5)
Other ^1^	5 (8)

OC—ovarian cancer, N/A—data not available, N—number of participants, BMI—body mass index, ^1^—cellulae carcinomatosae, adenocarcinoma solidum, infiltratio carcinomatosa ovarii, foliculoma, undifferentiated.

## Data Availability

The datasets used and analyzed during the current study are available from the corresponding author on reasonable request.

## Supplementary Materials

The following supporting information can be downloaded at: https://www.mdpi.com/article/10.3390/cancers14194899/s1, Table S1: Gene of interest mutual correlations in all participants and the case-control study. Table S2: Gene of interest mutual correlations in unchanged tissue and controls with benign, non-cancerous changes. Table S3: Gene of interest mutual correlations in tissue samples obtained from pre-menopausal and post-menopausal women. Table S4: Gene of interest mutual correlations in tissue samples obtained from women with comorbidities absence and presence. Figure S1. Box-whiskers plot of HIF1A, EPAS1, and VEGFA normalized expression level in controls and malignant tumors based on Youden’s *J* Index, the cut-off points for age, BMI, and comorbidities. Cr norm—normalized concentration ratio; * *p* < 0.05, ** *p* < 0.01, *** *p* < 0.001.

## References

[1] Jessmon P., Boulanger T., Zhou W., Patwardhan P. (2017). Epidemiology and treatment patterns of epithelial ovarian cancer. Expert Rev. Anticancer Ther..

[2] Torre L., Trabert B., DeSantis C., Miller K., Samimi G., Runowicz C., Gaudet M., Jemal A., Siegel R. (2018). Ovarian cancer statistics, 2018. CA Cancer J. Clin..

[3] Žilovič D., Čiurlienė R., Sabaliauskaitė R., Jarmalaitė S. (2021). Future Screening Prospects for Ovarian Cancer. Cancers.

[4] Fagotti A., Gallotta V., Romano F., Fanfani F., Rossitto C., Naldini A., Vigliotta M., Scambia G. (2010). Peritoneal carcinosis of ovarian origin. World J. Gastrointest. Oncol..

[5] Woopen H., Sehouli J. (2009). Current and Future Options in the Treatment of Malignant Ascites in Ovarian Cancer. Anti-Cancer Res..

[6] Osada R., Horiuchi A., Kikuchi N., Yoshida J., Hayashi A., Ota M., Katsuyama Y., Mellilo G., Konishi I. (2007). Expression of hypoxia-inducible factor 1α, hypoxia-inducible factor 2α, and von Hippel–Lindau protein in epithelial ovarian neoplasms and allelic loss of von Hippel-Lindau gene: Nuclear expression of hypoxia-inducible factor 1α is an independent prognostic factor in ovarian carcinoma. Hum. Pathol..

[7] Mutch D., Williams S. (1994). Biology of epithelial ovarian cancer. Clin. Obstet. Gynecol..

[8] Samulak D., Sajdak S., Wilczak M., Pieta B., Englert-Golon M. (2010). Evaluation of preoperative diagnosis with results of histopathological examinations of ovarian tumors in women of reproductive age - PubMed. Eur. J. Gynaecol. Oncol..

[9] Talks K., Turley H., Gatter K., Maxwell P., Pugh C., Ratcliffe P., Harris A. (2000). The expression and distribution of the hypoxia-inducible factors HIF-1alpha and HIF-2alpha in normal human tissues, cancers, and tumor-associated macrophages. Am. J. Pathol..

[10] Bunn H., Poyton R. (1996). Oxygen sensing and molecular adaptation to hypoxia. Physiol. Rev..

[11] Höckel M., Schlenger K., Höckel S., Aral B., Schäffer U., Vaupel P. (1998). Tumor Hypoxia in Pelvic Recurrences of Cervical Cancer. Int. J. Cancer.

[12] Stadler P., Becker A., Feldmann H.J., Hänsgen G., Dunst J., Würschmidt F., Molls M. (1999). Influence of the hypoxic subvolume on the survival of patients with head and neck cancer. Int. J. Radiat. Oncol. Biol. Phys..

[13] Zhang T., Suo C., Zheng C., Zhang H. (2019). Hypoxia and Metabolism in Metastasis. Adv. Exp. Med. Biol..

[14] Gómez-Raposo C., Mendiola M., Barriuso J., Casado E., Hardisson D., Redondo A. (2009). Angiogenesis and ovarian cancer. Clin. Transl. Oncol..

[15] Folkman J., Kalluri R., Kufe D.W., Pollock R.E., Weichselbaum R.R., Bast R.C.J., Gansler T.S., Holland J.F., Frei E.I. (2003). Tumor Angiogenesis. Holland-Frei Cancer Medicine.

[16] Liotta L.A., Stetler-Stevenson W.G. (1991). Tumor Invasion and Metastasis: An Imbalance of Positive and Negative Regulation. Cancer Res..

[17] Yamamoto S., Konishi I., Mandai M., Kuroda H., Komatsu T., Nanbu K., Sakahara H., Mori T. (1997). Expression of vascular endothelial growth factor (VEGF) in epithelial ovarian neoplasms: Correlation with clinicopathology and patient survival, and analysis of serum VEGF levels. Br. J. Cancer.

[18] Bryant C.S., Munkarah A.R., Kumar S., Batchu R.B., Shah J.P., Berman J., Morris R.T., Jiang Z.L., Saed G.M. (2010). Reduction of hypoxia-induced angiogenesis in ovarian cancer cells by inhibition of HIF-1 alpha gene expression. Arch. Gynecol. Obstet..

[19] An D., Banerjee S., Lee J.-M. (2021). Recent advancements of antiangiogenic combination therapies in ovarian cancer. Cancer Treat. Rev..

[20] Ali E.M., Sheta M., Abed M., Mohsen E. (2019). Elevated serum and tissue VEGF associated with poor outcome in breast cancer patients. AJM.

[21] Nascimento C., Gameiro A., Ferreira J., Correia J., Ferreira F. (2021). Diagnostic Value of VEGF-A, VEGFR-1 and VEGFR-2 in Feline Mammary Carcinoma. Cancers.

[22] Carmeliet P., Jain R.K. (2000). Angiogenesis in cancer and other diseases. Nature.

[23] Carmeliet P., Dor Y., Herbert J.M., Fukumura D., Brusselmans K., Dewerchin M., Neeman M., Bono F., Abramovitch R., Maxwell P. (1998). Role of HIF-1alpha in hypoxia-mediated apoptosis, cell proliferation and tumour angiogenesis. Nature.

[24] Marxsen J.H., Schmitt O., Metzen E., Jelkmann W., Hellwig-Bürgel T. (2001). Vascular endothelial growth factor gene expression in the human breast cancer cell line MX-1 is controlled by O2 availability in vitro and in vivo. Ann. Anat.-Anat. Anzeiger.

[25] Chen X., Liu J., He B., Li Y., Liu S., Wu B., Wang S., Zhang S., Xu X., Wang J. (2015). Vascular endothelial growth factor (VEGF) regulation by hypoxia inducible factor-1 alpha (HIF1A) starts and peaks during endometrial breakdown, not repair, in a mouse menstrual-like model. Hum. Reprod..

[26] Semenza G.L. (2000). HIF-1: Using two hands to flip the angiogenic switch. Cancer Metastasis Rev..

[27] Semenza G.L. (2003). Targeting HIF-1 for cancer therapy. Nat. Rev. Cancer.

[28] Jain S., Maltepe E., Lu M.M., Simon C., Bradfield C.A. (1998). Expression of ARNT, ARNT2, HIF1α, HIF2α and Ah receptor mRNAs in the developing mouse. Mech. Dev..

[29] Sowter H.M., Raval R., Moore J., Ratcliffe P.J., Harris A.L. (2003). Predominant role of hypoxia-inducible transcription factor (Hif)-1α versus Hif-2α in regulation of the transcriptional response to hypoxial. Cancer Res..

[30] Hu C.-J., Iyer S., Sataur A., Covello K.L., Chodosh L.A., Simon M.C. (2006). Differential Regulation of the Transcriptional Activities of Hypoxia-Inducible Factor 1 Alpha (HIF-1α) and HIF-2α in Stem Cells. Mol. Cell. Biol..

[31] Warnecke C., Zaborowska Z., Kurreck J., Erdmann V.A., Frei U., Wiesener M., Eckardt K. (2004). Differentiating the functional role of hypoxia-inducible factor (HIF)-1α and HIF-2α (EPAS-1) by the use of RNA interference: Erythropoietin is a HIF-2α target gene in Hep3B and Kelly cells. FASEB J..

[32] Keith B., Johnson R., Simon M.C. (2011). HIF1α and HIF2α: Sibling rivalry in hypoxic tumour growth and progression. Nat. Rev. Cancer.

[33] Al Tameemi W., Dale T., Al-Jumaily R., Forsyth N. (2019). Hypoxia-Modified Cancer Cell Metabolism. Front. Cell Dev. Biol..

[34] Peng S., Zhang J., Tan X., Huang Y., Xu J., Silk N., Zhang D., Liu Q., Jiang J. (2020). The VHL/HIF Axis in the Development and Treatment of Pheochromocytoma/Paraganglioma. Front. Endocrinol..

[35] Bertout J., Patel A.S., Simon M.C. (2008). The impact of O2 availability on human cancer. Nat. Rev. Cancer.

[36] Triner D., Shah Y. (2016). Hypoxia-inducible factors: A central link between inflammation and cancer. J. Clin. Investig..

[37] Wigerup C., Påhlman S., Bexell D. (2016). Therapeutic targeting of hypoxia and hypoxia-inducible factors in cancer. Pharmacol. Ther..

[38] Melillo G. (2006). Inhibiting hypoxia-inducible factor 1 for cancer therapy. Mol. Cancer Res..

[39] Englert-Golon M., Andrusiewicz M., Żbikowska A., Chmielewska M., Sajdak S., Kotwicka M. (2021). Altered Expression of ESR1, ESR2, PELP1 and c-SRC Genes Is Associated with Ovarian Cancer Manifestation. Int. J. Mol. Sci..

[40] Camacho-Sanchez M., Burraco P., Gomez-Mestre I., Leonard J.A. (2013). Preservation of RNA and DNA from mammal samples under field conditions. Mol. Ecol. Resour..

[41] Andrusiewicz M., Słowikowski B., Skibińska I., Wołuń-Cholewa M., Dera-Szymanowska A. (2016). Selection of reliable reference genes in eutopic and ectopic endometrium for quantitative expression studies. Biomed. Pharmacother..

[42] Janusz P., Chmielewska M., Andrusiewicz M., Kotwicka M., Kotwicki T. (2021). Methylation of Estrogen Receptor 1 Gene in the Paraspinal Muscles of Girls with Idiopathic Scoliosis and Its Association with Disease Severity. Genes.

[43] Skibińska I., Andrusiewicz M., Soin M., Jendraszak M., Urbaniak P., Jedrzejczak P., Kotwicka M. (2018). Increased expression of PELP1 in human sperm is correlated with decreased semen quality. Asian J. Androl..

[44] Englert-Golon M., Burchardt B., Budny B., Dębicki S., Majchrzycka B., Wrotkowska E., Jasiński P., Ziemnicka K., Słopień R., Ruchała M. (2017). Genomic markers of ovarian adenocarcinoma and its relevancy to the effectiveness of chemotherapy. Oncol. Lett..

[45] Gkotinakou I.M., Kechagia E., Pazaitou-Panayiotou K., Mylonis I., Liakos P., Tsakalof A. (2020). Calcitriol Suppresses HIF-1 and HIF-2 Transcriptional Activity by Reducing HIF-1/2α Protein Levels via a VDR-Independent Mechanism. Cells.

[46] Wierzbicki P.M., Klacz J., Kotulak-Chrzaszcz A., Wronska A., Stanislawowski M., Rybarczyk A., Ludziejewska A., Kmiec Z., Matuszewski M. (2019). Prognostic significance of VHL, HIF1A, HIF2A, VEGFA and p53 expression in patients with clear-cell renal cell carcinoma treated with sunitinib as first-line treatment. Int. J. Oncol..

[47] Hashimoto T., Shibasaki F. (2015). Hypoxia-Inducible Factor as an Angiogenic Master Switch. Front. Pediatr..

[48] Wong C., Wellman T.L., Lounsbury K.M. (2003). VEGF and HIF-1α expression are increased in advanced stages of epithelial ovarian cancer. Gynecol. Oncol..

[49] Birner P., Schindl M., Obermair A., Breitenecker G., Oberhuber G. (2001). Expression of hypoxia-inducible factor 1α in epithelial ovarian tumors: Its impact on prognosis and on response to chemotherapy. Clin. Cancer Res..

[50] Horiuchi A., Imai T., Shimizu M., Oka K., Wang C., Nikaido T., Konishi I. (2002). Hypoxia-induced changes in the expression of VEGF, HIF-1α and cell cycle-related molecules in ovarian cancer cells. Anti-Cancer Res..

[51] Bamberger E.S., Perrett C.W. (2002). Angiogenesis in epithelian ovarian cancer. J. Clin. Pathol. Mol. Pathol..

[52] Boocock C.A., Charnock-jones D.S., Sharkey A.M., Mclaren J., Barker P.J., Wright K.A., Twentyman P.R., Smith S.K. (1995). Expression of vascular endothelial growth factor and its receptors fit and KDR in ovarian carcinoma. J. Natl. Cancer Inst..

[53] Ceci C., Atzori M.G., Lacal P.M., Graziani G. (2020). Role of VEGFs/VEGFR-1 Signaling and Its Inhibition in Modulating Tumor Invasion: Experimental Evidence in Different Metastatic Cancer Models. Int. J. Mol. Sci..

[54] Horikawa N., Abiko K., Matsumura N., Hamanishi J., Baba T., Yamaguchi K., Yoshioka Y., Koshiyama M., Konishi I. (2017). Expression of Vascular Endothelial Growth Factor in Ovarian Cancer Inhibits Tumor Immunity through the Accumulation of Myeloid-Derived Suppressor Cells. Clin. Cancer Res..

[55] Beasley N.J.P., Leek R., Alam M., Turley H., Cox G.J., Gatter K., Millard P., Fuggle S., Harris A.L. (2002). Hypoxia-inducible Factors HIF-1 and HIF-2 in Head and Neck Cancer: Relationship to Tumor Biology and Treatment Outcome in Surgically Resected Patients. Cancer Res..

[56] Noguera R., Fredlund E., Piqueras M., Pietras A., Beckman S., Navarro S., Påhlman S. (2009). HIF-1alpha and HIF-2alpha are differentially regulated in vivo in neuroblastoma: High HIF-1alpha correlates negatively to advanced clinical stage and tumor vascularization. Clin. Cancer Res..

[57] Volm M., Koomägi R. (2000). Hypoxia-inducible factor (HIF-1) and its relationship to apoptosis and proliferation in lung cancer. Anti-Cancer Res..

[58] Greb R., Maier I., Wallwiener D., Kiesel L. (1999). Vascular endothelial growth factor A (VEGF-A) mRNA expression levels decrease after menopause in normal breast tissue but not in breast cancer lesions. Br. J. Cancer.

[59] Skrypnik D., Mostowska A., Jagodziński P.P., Bogdański P. (2020). Association of rs699947 (−2578 C/A) and rs2010963 (−634 G/C) Single Nucleotide Polymorphisms of the VEGF Gene, VEGF-A and Leptin Serum Level, and Cardiovascular Risk in Patients with Excess Body Mass: A Case–Control Study. J. Clin. Med..

[60] Zahid H., Subbaramaiah K., Iyengar N., Zhou X., Chen I., Bhardwaj P., Gucalp A., Morrow M., Hudis C., Dannenberg A. (2018). Leptin regulation of the p53-HIF1α/PKM2-aromatase axis in breast adipose stromal cells: A novel mechanism for the obesity-breast cancer link. Int. J. Obes..

[61] Giordano M., Boldrini L., Servadio A., Niccoli C., Melfi F., Lucchi M., Mussi A., Fontanini G. (2018). Differential microRNA expression profiles between young and old lung adenocarcinoma patients. Am. J. Transl. Res..

[62] Ganggaiswari A., Kresno S., Krisnuhoni E. (2010). VEGF expression and desmoplastic reaction as potential progressive factors in young patients with colorectal cancer. Acta Med. Indones..

[63] Nakayama K., Kanzaki A., Hata K., Katabuchi H., Okamura H., Miyazaki K., Fukumoto M., Takebayashi Y. (2002). Hypoxia-inducible factor 1 alpha (HIF-1α) gene expression in human ovarian carcinoma. Cancer Lett..

[64] Komatsu H., Oishi T., Itamochi H., Shimada M., Sato S., Chikumi J., Sato S., Nonaka M., Sawada M., Wakahara M. (2017). Serum Vascular Endothelial Growth Factor-A as a Prognostic Biomarker for Epithelial Ovarian Cancer. Int. J. Gynecol. Cancer.

[65] Zhou P., Xiong T., Chen J., Li F., Qi T., Yuan J. (2019). Clinical significance of melanoma cell adhesion molecule CD146 and VEGFA expression in epithelial ovarian cancer. Oncol. Lett..

[66] Petrella B.L., Brinckerhoff C.E. (2009). PTEN suppression of YY1 induces HIF-2α activity in von Hippel Lindau null renal cell carcinoma. Cancer Biol. Ther..

[67] Singhal R., Mitta S.R., Olive K.P., Lyssiotis C.A., Shah Y.M. (2019). Hypoxia inducible factor-2α increases sensitivity of colon cancer cells towards oxidative cell death. bioRxiv.

[68] English W., Lunt S., Fisher M., Lefley D., Dhingra M., Lee Y., Bingham K., Hurrell J., Lyons S., Kanthou C. (2017). Differential Expression of VEGFA Isoforms Regulates Metastasis and Response to Anti-VEGFA Therapy in Sarcoma. Cancer Res..

[69] Terme M., Pernot S., Marcheteau E., Sandoval F., Benhamouda N., Colussi O., Dubreuil O., Carpentier A., Tartour E., Taieb J. (2013). VEGFA-VEGFR pathway blockade inhibits tumor-induced regulatory T-cell proliferation in colorectal cancer. Cancer Res..

[70] Yang J., Yan J., Liu B. (2018). Targeting VEGF/VEGFR to Modulate Antitumor Immunity. Front. Immunol..

[71] García García Y., Marín Alcalá M., Martínez Vila C. (2020). Anti-angiogenic therapy for ovarian cancer. EJC Suppl..

