# Supplementary material for: Differential Expression of HIF1A, EPAS1, and VEGF Genes in Benign and Malignant Ovarian Neoplasia
Source: Cancers (Basel). 2022 Oct 7;14(19):4899. doi: 10.3390/cancers14194899 (PMC9563807; doi:10.3390/cancers14194899)
Supplement: Supplementary file 1 [file cancers-14-04899-s001.zip › cancers-1916581-supplementary.pdf]

Supplementary Materials

Differential Expression of HIF1A, EPAS1, and VEGF Genes in Benign and Malignant Ovarian Neoplasia

Monika Englert-Golon, Małgorzata Tokłowicz, Aleksandra Żbikowska, Stefan Sajdak, Małgorzata Kotwicka and Mirosław Andrusiewicz

Table S1. Gene of interest mutual correlations in all participants and the case-control study.

Group →	All participants (n = 114)		Control ovary (n = 59)		Malignant tumors (n = 55)	
Genes ↓	R	p-value	R	p-value	R	p-value
HIF1A & *EPAS1*	0.47	**<0.0001**	0.26	**0.0473**	0.57	**<0.0001**
HIF1A & *VEGFA*	0.25	**0.0077**	0.53	**<0.0001**	0.23	0.0899
EPAS1 & *VEGFA*	0.09	0.3395	0.36	**0.0056**	0.15	0.2817

N – number of cases; R – Spearman's rank correlation coefficient; p-values < 0.05 are indicated in bold.

Table S2. Gene of interest mutual correlations in unchanged tissue and controls with benign, non-cancerous changes.

Group →	Ovary without changes (n = 38)		Benign ovarian change (n = 21)	
Genes ↓	R	p-value	R	p-value
HIF1A & *EPAS1*	0.32	**0.0474**	0.17	0.4702
HIF1A & *VEGFA*	0.71	**<0.0001**	0.08	0.7237
EPAS1 & *VEGFA*	0.45	**0.0045**	0.08	0.7364

N – number of cases; R – Spearman's rank correlation coefficient; p-values < 0.05 are indicated in bold.

Table S3. Gene of interest mutual correlations in tissue samples obtained from pre-menopausal and post-menopausal women.

Group →	Pre-menopausal cases (n = 34)		Post-menopausal cases (n = 77)	
Genes ↓	R	p-value	R	p-value
HIF1A & *EPAS1*	0.27	0.1211	0.50	**<0.0001**
HIF1A & *VEGFA*	0.47	**0.0053**	0.16	0.1705
EPAS1 & *VEGFA*	0.17	0.3284	0.12	0.3189

N – number of cases; R – Spearman's rank correlation coefficient; p-values < 0.05 are indicated in bold.

Table S4. Gene of interest mutual correlations in tissue samples obtained from women with comorbidities absence and presence.

Group →	Comorbidities absence (n = 37)		Comorbidities presence (n = 67)	
Genes ↓	R	p-value	R	p-value
HIF1A & *EPAS1*	0.34	**0.0404**	0.40	**0.0007**
HIF1A & *VEGFA*	0.32	0.0567	0.15	0.2194
EPAS1 & *VEGFA*	−0.05	0.7488	0.03	0.7905

N – number of cases; R – Spearman's rank correlation coefficient; p-values < 0.05 are indicated in bold.

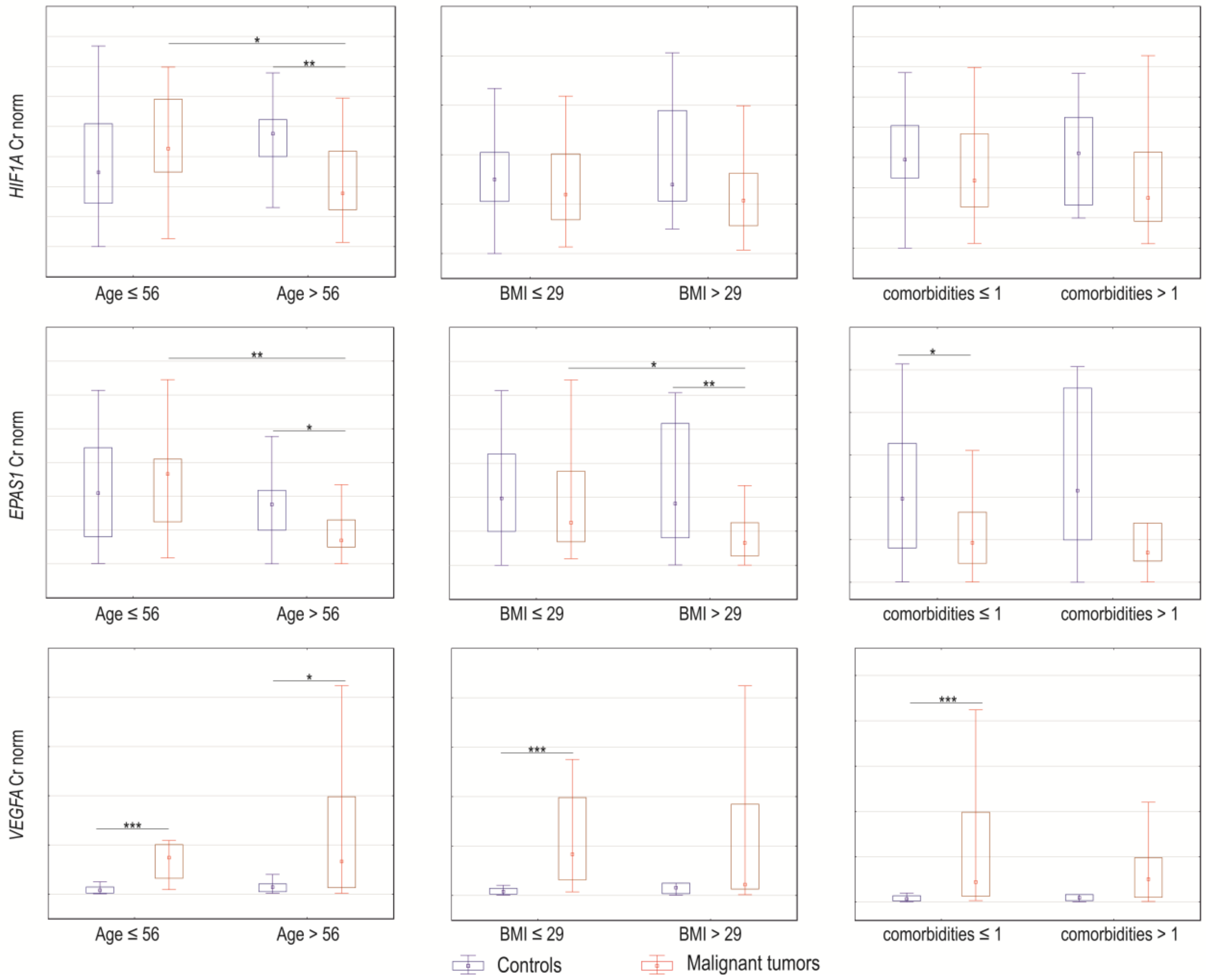

Figure S1. Box-whiskers plot of *HIF1A, EPAS1,* and *VEGFA* normalized expression level in controls and malignant tumors based on Youden's *J* Index, the cut-off points for age, BMI, and comorbidities. Cr norm – normalized concentration ratio; * $p < 0.05$, ** $p < 0.01$, *** $p < 0.001$.
